# A fundamental limit to the effectiveness of traveller screening with molecular tests

**DOI:** 10.1017/S0950268825100381

**Published:** 2025-08-15

**Authors:** Kate Bubar, Casey Middleton, Daniel Larremore, Katelyn Gostic

**Affiliations:** 1 Department of Computer Science, University of Colorado Boulder, Boulder, CO, USA; 2 BioFrontiers Institute, University of Colorado Boulder, Boulder, CO, USA; 3 Santa Fe Institute, Santa Fe, NM, USA; 4 Department of Ecology and Evolution, University of Chicago, Chicago, IL, USA

**Keywords:** traveller screening, molecular diagnostics, probabilistic model, viral kinetics, COVID-19 testing

## Abstract

Despite the appeal of screening travellers to prevent case importation during infectious disease outbreaks, evidence shows that symptom screening is largely ineffective in delaying the geographical spread of infection. Molecular tests offer high sensitivity and specificity and can detect infections earlier than symptom screening, suggesting potential for improved outcomes. However, they were used to screen travellers for COVID-19 with mixed success. To investigate molecular screening’s role in controlling COVID-19, and to quantify the effectiveness of screening for future pathogens of concern, we developed a probabilistic model that incorporates within-host viral kinetics. We then evaluated the potential effectiveness of screening travellers for influenza A, SARS-CoV-1, SARS-CoV-2, and Ebola virus. Even under highly optimistic assumptions, we found that the inability to detect recent infections always limits the effectiveness of traveller screening. We quantify this fundamental limit by proposing an estimator for the fraction of transmission that is preventable by screening. We also demonstrate that estimates of ascertainment overestimate reductions in transmission. These results highlight the essential role that quarantine and repeated testing play in infectious disease containment. Furthermore, our findings indicate that improving screening effectiveness requires the ability to detect infection much earlier than current state-of-the-art molecular tests.

## Introduction

Screening travellers at airports is a common countermeasure used to prevent or delay the geographical spread of infection during an infectious disease outbreak. However, scenario modelling [1–5] and overwhelming empirical evidence [6–10] show that syndromic and questionnaire-based screening programmes are typically ineffective. Novel rapid molecular tests could be more effective than other screening methods because of their high sensitivity and specificity over a long detectable window with rapid turnaround.

While molecular tests were used to screen travellers during the COVID-19 pandemic, the jurisdictions that most successfully prevented or delayed transmission of SARS-CoV-2 such as New Zealand, Australia, Hong Kong, and Taiwan also had strict border controls, post-arrival quarantine measures, widespread testing, or contact tracing. As a result, it is unclear what role molecular testing of travellers per se played in practice, and what little evidence we do have (reviewed in [11]) reports only the number of individuals screening positive, but not the programmes’ effectiveness in delaying transmission.

In place of empirical data, modelling studies offer various estimates of traveller screening effectiveness for SARS-CoV-2 in particular, analysing different aspects of testing programmes like the benefits of PCR versus rapid diagnostic tests (RDTs) [12–15]. In summary, screening with currently available molecular tests was found to be ineffective in meaningfully delaying the spread of SARS-CoV-2 in previously unaffected areas, despite their high sensitivity.

We aim to understand when screening travellers for pathogens of concern with state-of-the-art molecular tests may effectively prevent or delay an outbreak at the travellers’ destination.

## Methods

### Model for traveller screening

From a public health perspective, the most important infections for a traveller screening programme to catch are those most likely to infect others during or after travel, and the least important are those with little to no remaining infectiousness. To incorporate this concept into a mathematical model, we considered an individual’s post-travel transmission potential, denoted as 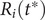 . 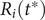 is the expected number of secondary infections generated by individual 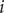 after travelling at time 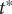 . Mathematically,

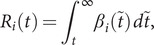

where 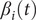 is an individual’s infectiousness during their course of infection. Here we assume 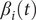 reflects time-varying infectiousness with constant social behaviour and no interventions, but it would be possible to extend the model so that 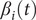 incorporates variation in all three factors. This approach accounts for variation in individual reproductive numbers and infection age at the time of travel.

**Figure 1.**
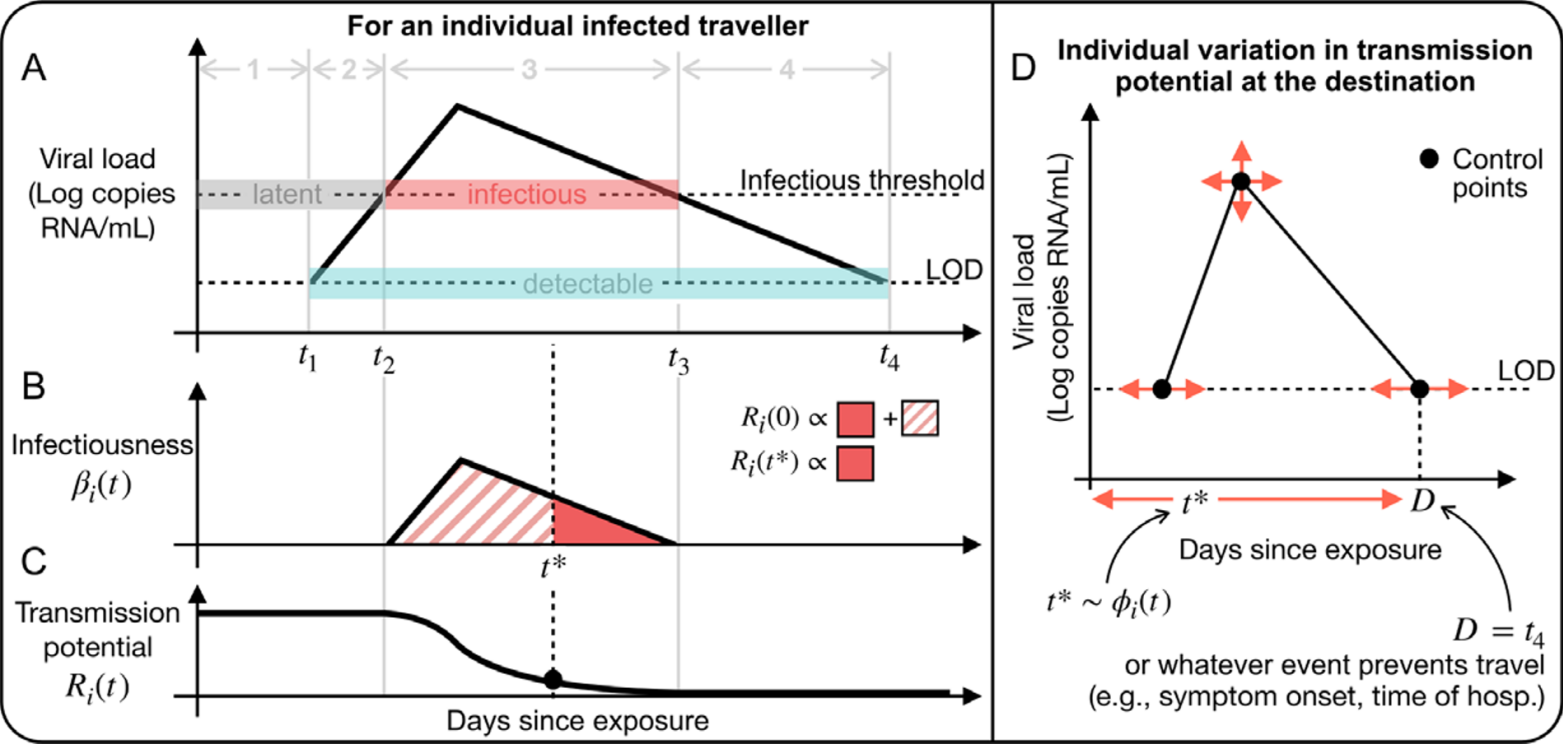
Model diagram. An example (a) viral load, (b) infectiousness 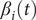 , and (c) transmission potential 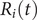 for an individual infected traveller 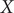 , with travel time 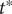 and post-travel transmission potential 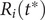 . There are four possible statuses for infected travellers: (1) not yet detectable or infectious, (2) detectable and not yet infectious, (3) detectable and infectious, and (4) detectable and no longer infectious. (d) Factors that contribute to variation in 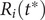 : Stochastic realizations of viral load control points (first and last time detectable, peak viral load), when people may travel 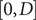 , and the simulated travel time 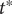 drawn from 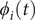 , the infection age distribution among infected travellers.

To compute 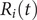 , we approximate 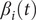 using a simple within-host viral kinetics model (Figure 1a,b). We assume there is a period after infection where the virus is undetectable, followed by a proliferation phase of exponential growth and then a clearance phase of exponential decay. This type of log-linear model, also referred to as a hinge or tent function, is commonly used to model viral infection [16–20]. We assume infectiousness 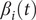 is proportional to log viral loads above an infectious threshold. This model makes 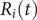 a monotonically decreasing function, which is biologically and mathematically realistic: the number of expected secondary transmission events ahead in time decreases as an individual’s infection progresses (Figure 1c).

Throughout this work, we intentionally make optimistic assumptions about test performance, assuming instantaneous test results, perfect compliance, and a limit of detection (LOD) equal to that of RT-PCR (hereafter PCR), the gold standard LOD currently achievable for the diseases in this study. We assumed 100% sensitivity above the LOD. For currently available technology, these assumptions are unrealistic because there is a trade-off between sensitivity and turnaround time [19]. However, these optimistic assumptions allow us to characterize the best-case scenario, and thus the *potential* effectiveness of screening programmes, with currently achievable test LODs.

We considered screening only at points of exit, rather than paired screening at points of exit and entry. Prior work has found little marginal benefit for an additional test at points of entry, provided that the screening method is highly sensitive [1, 3]. We also assumed that the outbreak at the departure location is in a phase of exponential growth, an assumption relevant to screening-based containment scenarios, and one which affects the demographic distribution of infection ages among those attempting travel.

### Quantifying screening effectiveness

We considered two different approaches to quantify traveller screening effectiveness. First, we considered how many additional infected travel attempts could be tolerated before causing an outbreak with high probability in screening vs. no-screening scenarios ( 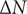 *).* To calculate 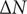 , we used theory from stochastic processes about the long-term probability of extinction to compute the number of infected travellers required to cause an outbreak with probability 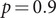 . Second, we considered how much longer it takes for an outbreak of size 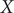 to occur at the destination in screening vs. no-screening scenarios ( 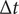 ). To calculate 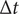 , we assumed the number of infected travellers arriving at the airport followed a Poisson process with mean 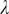 infected travellers per day. We simulated transmission chains initialized by infected travellers at the destination until 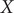 infections had occurred. See Supplementary Sections S1 and S2 for more details.

### Simulations

To simulate an individual infected traveller, we sampled a time they are first and last detectable by a molecular test with a PCR LOD, a time and magnitude of peak viral load, and a time of hospitalization, if applicable, from the distributions in Supplementary Table S2. When well-characterized distributions were not available, we used optimistic estimates, in terms of potential screening effectiveness, informed by existing literature. The infection age distribution among infected travellers, 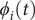 , is a mixture of the infection age distribution and the propensity to travel at a particular age. Individuals’ travel times 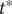 are sampled from 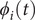 using the inverse CDF method. See Supplementary Sections S4 and S3 for more details. With these parameters, we compute individuals’ 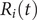 and screening result at the time of travel.

For each individual, we simulate their contribution to infection at the destination using a branching process in which the offspring distribution of the first generation is a Poisson distribution with 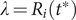 and for subsequent generations, a Negative binomial distribution with mean 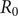 and dispersion parameter 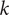 [21]. If 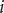 is not detected, the simulated branching processes are identical with and without screening. This approach of comparing counterfactual scenarios ensures our results reflect the impact of screening alone and not the stochasticity of transmission.

## Results

### Screening effectiveness to delay transmission

**Figure 2.**
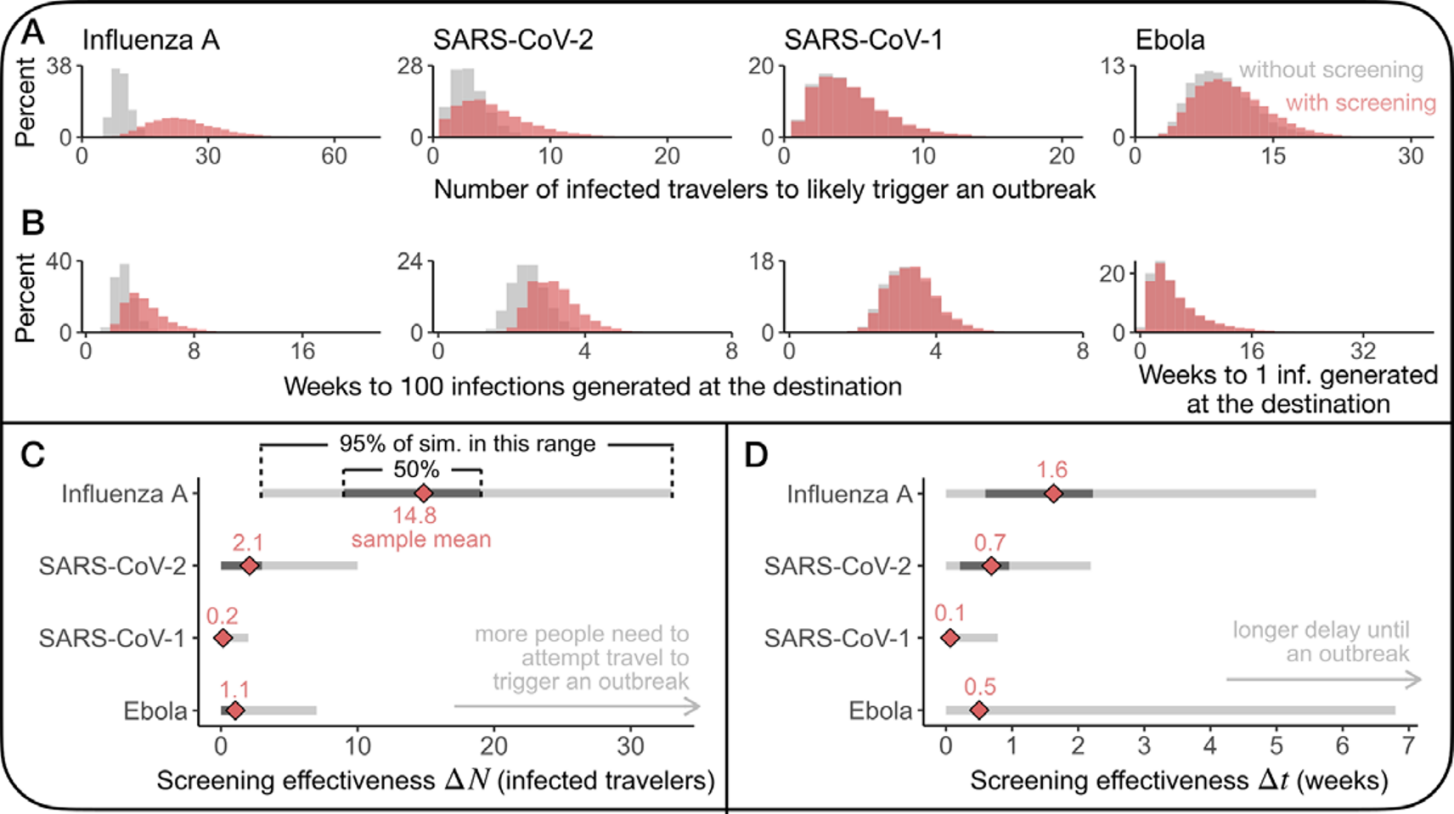
Screening effectiveness to delay transmission is limited and highly variable. Histograms of (a) the number of infected travellers to likely trigger an outbreak with (pink) and without screening (grey) and (b) the time to 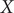 infections generated at the destination with (pink) and without screening (grey) from 20000 Monte Carlo simulations. 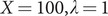 per day for SARS-CoV-1, SARS-CoV-2, and influenza A. 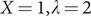 per month for Ebola. (c, d) Distributions of 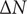 and 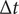 from 20000 Monte Carlo simulations (sample mean (pink diamond), IQR (dark grey) and 95% percentile range (light grey)).

We simulated 5000 infected travellers for four pathogens: SARS-CoV-1, SARS-CoV-2, influenza A, and Ebola. We chose these pathogens because their natural histories probe different areas of the parameter space of our model, and because traveller screening programmes have been implemented for all of them. Then, we ran 20000 simulations of the travelling process with and without screening in place (Figure 2a,b). To calculate the time until an outbreak of size 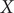 , we constructed one scenario representing importations from a rapidly spreading acute respiratory infection (for SARS-CoV-1, SARS-CoV-2, and influenza A, 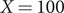 infections and 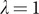 infected traveller per day) and another representing importations from a smouldering haemorrhagic fever outbreak in which even one transmission event at the destination would cause tremendous concern (for Ebola, 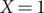 infection and 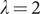 per month). Of the four pathogens we considered, traveller screening is most effective for influenza A. With screening in place, it takes an average of 15 more infected individuals to attempt travel to trigger an outbreak in comparison to no screening programme (Figure 2c). In units of time, screening delayed an influenza A outbreak at the destination by 11.1 days on average (Figure 2d). However, there is considerable variation in both outcomes, as shown by the range of the central 50% of simulations (the interquartile range) (Figure 2c,d). Another way to understand this variation is to compute the probability that screening delayed an outbreak by at least 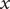 infected travellers or 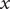 days. For example, screening delayed an outbreak by at least 1 week in 57.6% of simulations for influenza A. See Supplementary Table S1 for more values of 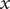 .

Traveller screening was less effective for the other three pathogens. On average screening allowed for 0.2, 1.1, and 2.1 additional infected travel attempts before an outbreak likely occurred for SARS-CoV-1, Ebola, and SARS-CoV-2, respectively. In units of time, screening delayed outbreaks by 0.4, 3.5, and 4.8 days on average for SARS-CoV-1, Ebola, and SARS-CoV-2, respectively (Figure 2d). Once again, it is important to consider the variation in these outcomes. For example, although screening typically delayed an outbreak of Ebola by 3.5 days, in over 50% of simulations, there is no delay at all (Figure 2d). Screening delayed an outbreak by at least a week in only 1.4%, 9.3%, and 23.4% of simulations for SARS-CoV-1, Ebola, and SARS-CoV-2, respectively.

### Fundamental limit of traveller screening

**Figure 3.**
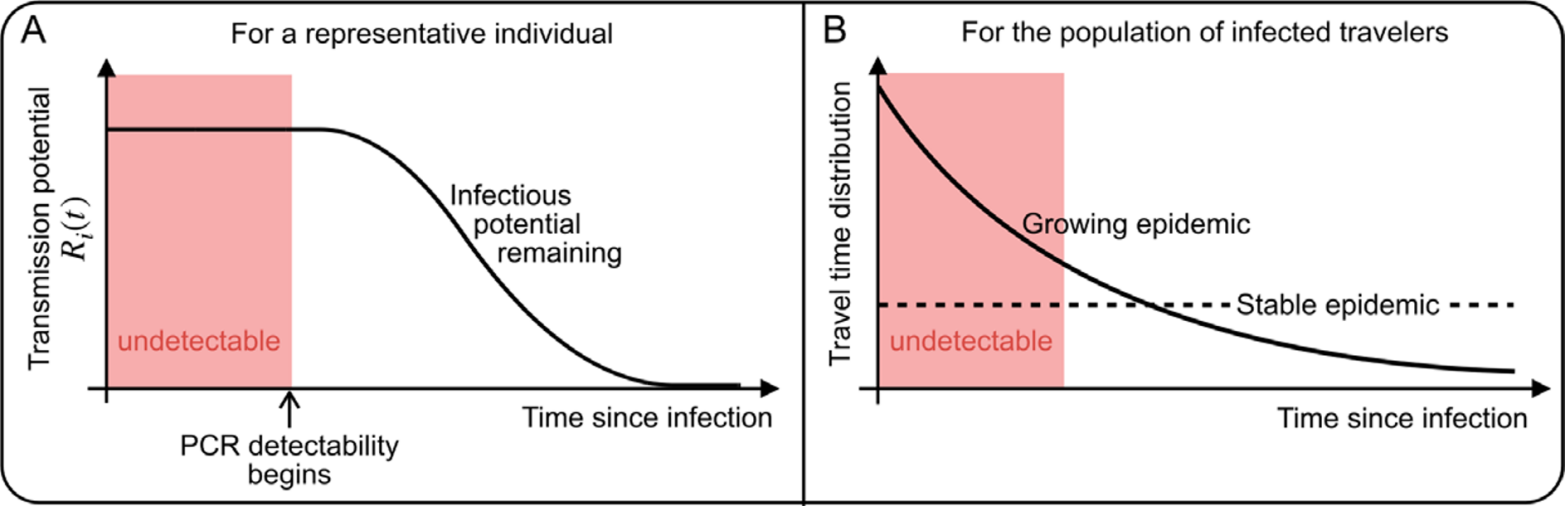
The effectiveness of screening travellers is fundamentally limited by the gap between infection and detectability. (a) Individuals are undetectable by molecular testing when their transmission potential is highest, fundamentally limiting the effectiveness of traveller screening because infected people may travel during this window. (b) A growing epidemic exacerbates this fundamental limit because the infection age distribution among infected travellers is positively skewed in comparison to a stable epidemic.

In all tested scenarios, under optimistic assumptions about detectability and test sensitivity, the simulated effectiveness of screening varied greatly, and often screening did not delay transmission at all (Figure 2c,d). To understand this result, we observe that, for any test or pathogen, there always exists a gap between when someone is infected and first detectable. This implies that there is a window of time when an infected individual is undetectable and may travel. Crucially, travellers who are missed by screening during this window have all their transmission potential remaining (Figure 3a). Thus, the travellers with the most transmission potential are impossible to catch.

To quantify how this window of time limits traveller screening effectiveness, we calculated the expected proportion of transmission potential that is detectable by screening,

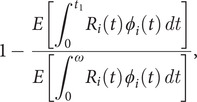

where 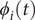 is the infection age distribution among infected travellers. Following the notation from Figure 1, 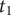 is the time individual 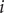 is first detectable, and 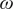 is the time 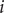 is either no longer infectious or no longer able to travel ( 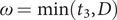 , see Supplementary Section S4 for more details).

Because of the gap between infection and detectability, the fraction in the expected proportion of transmission potential that is detectable by screening is always positive. Consequently, traveller screening alone can never eliminate the risk of local transmission at the travel destination, and this expression represents a fundamental limit to the effectiveness of traveller screening. The consequences of this fundamental limit are exacerbated during a growing epidemic, precisely when traveller screening programmes would likely be implemented, because the infection age distribution is skewed towards recent infections (Figure 3b). We found that 2.8%, 9.7%, 40.2%, and 59.8% of transmission potential is expected to be detectable by traveller screening via molecular test for SARS-CoV-1, Ebola, SARS-CoV-2, and influenza A, respectively. Our estimate for SARS-CoV-2 is comparable to other modelling studies (29–53%) [15, 14].

### Ascertainment overestimates transmission reduction

Previous studies have estimated the ascertainment rate of infected travellers as a measure of screening effectiveness [1, 2, 5]. Our study found that ascertainment is extremely low for SARS-CoV-1 and Ebola (3.1% and 10.5%, respectively), and better but still imperfect for SARS-CoV-2 and influenza A (47.8% and 70.9%, respectively). Our estimate of SARS-CoV-2 ascertainment is comparable to an empirical estimate from testing at U.S. airports during 2022 (52%) [22].

Ascertainment is a misleading substitute for screening effectiveness as a containment measure, because it overestimates reductions in transmission at the destination. This is because the typical undetected traveller has a greater post-travel transmission potential 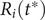 than the typical detected traveller: the average 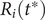 among undetected travellers is 2.6, 1.8, 2.5, and 1.2 for SARS-CoV-1, Ebola, SARS-CoV-2, and influenza A, respectively, while for detectable travellers, it is 2.8, 1.7, 1.8, and 0.8. This pattern occurs because many detected travellers are near the end of their infection and have little to no transmission potential (Supplementary Figure S1). Moreover, for all four pathogens, the per cent of post-travel transmission potential that is detectable by screening is always less than the corresponding ascertainment rate.

### Sensitivity analysis

We performed additional sensitivity analyses to explore how screening effectiveness varies across different epidemic scenarios, test characteristics, infectiousness profiles, and travelling behaviours.

We intuitively found that screening delays outbreaks for longer when infected people travel less frequently or when the outbreak threshold is larger (Supplementary Figures S2, S3, S4, and S5). However, even in the best-case scenario we considered ( 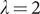 per month, 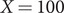 infections), screening delays an influenza A outbreak by less than 1 week in 38.9% of simulations (Supplementary Figure S2). Infectious thresholds are estimated in the literature for SARS-CoV-2 and influenza A but not for SARS-CoV-1 or Ebola. For these pathogens, we chose infectious thresholds so the distributions of individual reproductive numbers 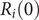 are similar to the gamma distribution with mean 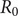 and dispersion parameter 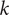 [21]. We ran sensitivity analyses with thresholds 10x larger and 10x smaller (Supplementary Figure S6) and found the change in the average 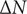 and 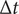 was small, at most 1 person or 2.4 days (Supplementary Figures S7, S8, S9, and S10).

Finally, we assumed the probability that an individual travels is uniform from infection to viral clearance for SARS-CoV-2 and influenza A. For SARS-CoV-1 and Ebola, we assumed symptoms prevent travel, limiting travel from infection to hospitalization. If symptom severity did not impede travel, the mean 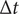 increased from 3.5 to 7.9 days for Ebola (Supplementary Figure S11) and from 0.5 to 3.8 days for SARS-CoV-1 (Supplementary Figure S12).

## Discussion

This study modelled the potential effectiveness of traveller screening programmes with highly sensitive molecular tests to delay transmission at the destination. Overall, we found that screening effectiveness is often negligible, or at best, highly variable. Of the four pathogens we considered, traveller screening was most effective for influenza A, but even under our optimistic assumptions about test performance, over 40% of post-travel transmission potential is *not* preventable by screening.

We derived an equation to quantify the fraction of transmission that is preventable by screening, which we show is never equal to zero, indicating a fundamental limit to traveller screening’s effectiveness for outbreak containment. The idea is simple: the effectiveness of traveller screening programmes will always be limited because, for every diagnostic test and pathogen, the newest infections with the most remaining transmission potential are impossible to catch. Even with state-of-the-art tests where people are detectable before they are infectious, there is a window of time after the infection event when individuals are not yet detectable and may travel, and their infectious period will not begin until they are at the destination. Thus, quarantine with repeated molecular testing for the duration of infectiousness [15], or in the case of syndromic screening, for the full incubation period [23], is essential for containment.

The fundamental limit equation indicates that the best-case scenario for effective screening would be a test that enables early detectability (ideally within hours [23]) and has near-zero turnaround time or if undetectable infections have low post-travel transmission potential. Importantly, this notion of controllability differs from effective control of community transmission which requires that detectability precede infectiousness [20, 24]. While this requirement is necessary for effective traveller screening, there also needs to be no undetectable window after infection.

While molecular tests are more sensitive than syndromic screening, they are not always superior for traveller screening. For example, we found screening via molecular tests is extremely ineffective for SARS-CoV-1, even though it is considered a controllable pathogen [24]. This discrepancy is due to symptom onset occurring before infectiousness and typically before detectability by PCR. In the initial days post symptom-onset, 50–80% of infections are negative by PCR with nasopharyngeal aspirate samples [25–30], possibly because viral replication starts in the lower respiratory tract [25]. Thus, the most effective screening method depends on the natural history of the disease [1].

This work assumes the goal of traveller screening is to prevent or delay local transmission at the destination. If local transmission is already underway, airport screening cannot prevent it and will have little to no effect on delaying a local outbreak. Screening programmes may have other goals such as general surveillance and public awareness of an ongoing outbreak. For example, in Venezuela in 2021, the introduction of the SARS-CoV-2 Omicron variant was rapidly detected in samples from airport screening [31].

Our findings are subject to several limitations. First, the data available to parameterize viral load trajectories for pathogens other than SARS-CoV-2 were limited. We used the best estimates available or extrapolated plausible ranges from other available information to characterize the control points in our study (See Supplementary Section S4 for more detail). Additionally, this model assumes all variation in individuals’ transmission potential is due to their viral load. While the use of log viral load as a proxy for infectiousness is supported in the literature (SARS-CoV-1 [24, 32], SARS-CoV-2 [33, 34], Ebola [35], influenza A [36]) and has been used in other modelling studies used [20, 37], other relationships between viral load and infectiousness [19, 38], other proxies for infectiousness [39, 40], or including changes in behaviour in the functional form of infectiousness are possible.

We assumed tests have a highly sensitive PCR LOD, around 10^2.6^ copies of RNA/mL (Supplementary Table S2). One way to improve screening effectiveness would be to design tests with a lower LOD so individuals are detectable earlier. Lower PCR limits of detection are physically possible, for example, by using more sensitive PCR enzymes or optimizing the PCR conditions, but reliability at lower thresholds is challenging. In practice, any operational delays in sample-to-answer times would substantially lower screening effectiveness, so our results, which assumed instantaneous results, represent an upper bound on the potential effectiveness of traveller screening.

Traveller screening programmes are typically expensive and resource-intensive to implement. Our results suggest that, while traveller screening may delay an outbreak at the destination, combining traveller screening with other interventions is necessary to more consistently delay, or ideally prevent, an outbreak post-travel. Unfortunately, screening travellers with more sophisticated rapid molecular diagnostics will not be as effective as hoped at delaying transmission because the travellers with the highest transmission potential are likely impossible to detect.

## Supporting information

10.1017/S0950268825100381.sm001 Bubar et al. supplementary material Bubar et al. supplementary material

## Data Availability

All code used in these analyses can be found at https://github.com/kbubar/travelerscreening.

## Supplementary material

The supplementary material for this article can be found at http://doi.org/10.1017/S0950268825100381.
