# Supplementary material for: A fundamental limit to the effectiveness of traveller screening with molecular tests
Source: Epidemiol Infect. 2025 Aug 15;153:e95. doi: 10.1017/S0950268825100381 (PMC12394024; doi:10.1017/S0950268825100381)
Supplement: Bubar et al. supplementary material [file S0950268825100381sup001.docx]

Supplemental Materials to

“A fundamental limit to the effectiveness of traveller

screening with molecular tests”

**S1 Number required to likely trigger an outbreak**

Following Clifford et. al. [[1](#_bookmark62)], we can estimate the long-term probability of disease extinction *s*_0_, for a negative binomial offspring distribution with mean *R*_0_ and dispersion parameter *k*, from the implicit equation

$$s_{0} =\left( 1 + \frac{R_{0}}{k}\left( 1-s_{0} \right) \right)^{-k}$$

Let $s_{i}$ be the long-term probability of disease extinction in a population where the first generation of infections is caused by infected traveller $i$with transmission potential $R_{i}\left( t^{*} \right)$, with offspring distribution Poisson($R_{i}\left( t^{*} \right)$), and each subsequent generation follows NegBinom${(R}_{0}, k)$. Then,

$$s_{i}=P\left( Z=0 \right)+P\left( Z=1 \right)s_{0}+P\left( Z=2 \right){s_{0}}^{2}+P\left( Z=3 \right){s_{0}}^{3}+\ldots$$

$${\to s}_{i}=e^{-R_{i}\left( t^{*} \right)}+R_{i}\left( t^{*} \right)e^{-R_{i}\left( t^{*} \right)}s_{0}+{\frac{{R_{i}\left( t^{*} \right)}^{2}}{2!}e}^{-R_{i}\left( t^{*} \right)}{s_{0}}^{2}+\frac{{R_{i}\left( t^{*} \right)}^{3}}{3!}e^{-R_{i}\left( t^{*} \right)}{s_{0}}^{3}+\ldots$$

$$\to s_{i}= e^{-R_{i}\left( t^{*} \right)(1-s_{0})}$$

The probability that infected traveller $i$ causes an outbreak at the destination is $q_{i}=1-s_{i}$, as in [[1](#_bookmark62)].

To calculate $N$, the number of infected travellers required to trigger an outbreak, we know that the first $N-1$ travellers did not cause an outbreak. If $q_{i}=q$ for all infected travellers, $X \sim\text{geometric}(q)$ so

$P\left( X\leq k \right)=1-{(1-q)}^{k}$.

However, since $q_{i}$ is dependent on an individual’s $R_{i}\left( t^{*} \right)$, $q_{i}$ is a random variable so

$$P\left( X\leq n \right)=1-\left( 1 - q_{1} \right)\left( 1 - q_{2} \right)\ldots(1 - q_{n})$$

for $n$ infected travellers. For each run of our model simulation, we can use this equation to compute the number of infected travellers $N$ required to cause an outbreak with probability $p$. For our analyses, we set $p=0.9$.

**S2 Time to** $\boldsymbol{X}$ **infections generated at the destination**

To compute this outcome via simulation, we first generate an arrival time for an infected traveller and simulate any transmission chains they generated using the distributions described above. We store the first $X$ subsequent cases and the timing of infection using the pathogen-specific generation interval. Then, we generate the arrival time of the next infected traveller. If this infected traveller arrived before the last stored case, or the number of cases at the destination is less than $X$, we repeat these steps until the requirements have been met. The output, time to $X$ infections generated at the destination is the time of the $X^{th}$ infection.

**S3 Infected travellers’ infection age distribution**

We assume traveller screening programs would be implemented at the beginning of an emerging infectious disease outbreak when infections are growing exponentially. So, as previously described in [[2](#_bookmark63)], the probability that an infected traveller has infection age $t$ at the time of travel is

$$\phi\left( t \right)=\left\{ \begin{aligned} \frac{\frac{R_{0}}{D}e^{\frac{-R_{0}}{D} t}}{1-e^{-R_{0}}}, &t\in[0, D] \\ \\ 0, &t>D \end{aligned} \right.$$

where $D$ is the duration of infection in which an infected individual is assumed to travel. If the disease does not prevent someone from travelling, $D$ is the time from infection to viral clearance. If symptoms prevent an individual from travelling, we assume $D$ is the average time from infection to hospitalization.

The corresponding CDF is the probability that an infected traveller was infected less than or equal to $t$ days before travel,

$$F\left( t \right)=\left\{ \begin{aligned} \frac{1-e^{\frac{-R_{0}}{D} t}}{1-e^{-R_{0}}}, &t\in[0, D] \\ \\ 0, &t>D. \end{aligned} \right.$$

**S4 Model parameterization**

Table [S2](#_bookmark51) contains all the parameter values and distributions used to simulate infected travellers for each pathogen. For each parameter that is treated as a random variable, we used the reported distribution when reported in the literature. If we could not find a reported distribution, we used a truncated normal when a mean and standard deviation were reported, truncated either at the lowest and highest reported values or within a reasonable range that captured most measurements. If mean and SD were not reported, then we used a uniform distribution within a reasonable range that captured most measurements. We used the serial interval as an approximation for the generation interval when generation interval estimates were not available. Examples of 100 simulated viral load trajectories for these four pathogens are shown in Supp. Fig. [S13](#_bookmark61). We chose the lowest reported PCR limit of detection, since this corresponds to a best-case scenario for testing. Below we elaborate on specific assumptions and rationale for each pathogen.

For SARS-CoV-2 and influenza A, we found estimates for the infectious threshold in the literature (Table [S2](#_bookmark51)). For SARS-CoV-1 and Ebola, we estimated infectious thresholds to result in distributions of $R_{i}(0)$ similar to the gamma distribution with mean $R_{0}$ and dispersion parameter $k$, a typical choice for the distribution of individual reproductive numbers [[3](#_bookmark64)]. Fitting the infectious threshold directly to the gamma distribution would assume that all the variation in individual reproductive numbers is due to differences in viral loads. However, we know that other factors contribute to differences in individual reproductive numbers so we would not expect the distribution of $R_{i}(0)$ from the viral load model to identically match the distribution of $R_{i}(0)$ fit to contact tracing data. We checked how sensitive our results were to the infectious threshold value in the sensitivity analyses (Supp. Fig. [S6](#_bookmark54), [S7](#_bookmark55), [S8](#_bookmark56), [S9](#_bookmark57), [S10](#_bookmark58)).

**SARS-CoV-1**

We assumed that all the cases are hospitalized. This is appropriate because hospitalization rates for symptomatic SARS-CoV-1 were high and, while estimates of asymptomatic infections vary from 0.1% [[4](#_bookmark65)] to 13% [[5](#_bookmark66)], there is no known transmission from asymptomatic patients so we do not consider them in our analyses [[5](#_bookmark66)]. We chose the distribution uniform(0,9) days post symptom onset as an optimistic guess for the time first detectable by PCR. This range was chosen based off data that reported 50-80% did not test positive via PCR in initial days post symptom onset [[6](#_bookmark67), [7](#_bookmark68), [8](#_bookmark69), [9](#_bookmark70), [10](#_bookmark71), [11](#_bookmark72)], *>*50% were positive by day 6-7 [[8](#_bookmark69), [7](#_bookmark68)], and *>*95% are PCR positive by day 10 [[10](#_bookmark71)]. Although not necessary for the model, we also parameterized individuals’ time of symptom onset since other parameters were measured in units of the time since symptom onset.

**SARS-CoV-2**

We parameterized the model for the ancestral strain of SARS-CoV-2. Many of the parameters needed for our model are well characterized by Kissler et. al. [[12](#_bookmark73)]. We did not distinguish between symptomatic and asymptomatic cases, nor did we include hospitalization.

**Influenza A**

Most references reported data from influenza H1N1 subtype, a few from influenza H3N2, and some simply referenced influenza A without specifying the subtype. We did not distinguish between symptomatic and asymptomatic cases, nor did we include hospitalization.

**Ebola**

For Ebola, higher viral load is correlated with mortality [[13](#_bookmark74)]. We parameterized the model for non- fatal cases assuming they would be more likely to travel and assumed all such cases are hospitalized. Although not necessary for the model, we parameterized individuals’ time of dry and wet symptom onset since other parameters were measured in units of the time since symptom onset. Note that the model parameters are not as well characterized for Ebola as other pathogens, possibly because the incubation and infectious periods are highly variable [[14](#_bookmark75)].

Our model of infectiousness implicitly assumes that we are only considering direct transmission via fomites, droplets, or aerosols [[15](#_bookmark76)], and not post-mortem transmission. Thus, we did not consider asymptomatic cases because they would not have transmission potential, and asymptomatic cases are rare [[16](#_bookmark77)].

We chose the distribution uniform(0, 3) days post onset of dry symptoms as an optimistic guess for the time first detectable by PCR. This range implies that individuals are sometimes detectable when they have dry symptoms, and everyone is detectable by the time their symptoms progress to wet symptoms. This was informed by the notion that there is no evidence that infected people are viraemic before symptom onset, but some are PCR positive on the day of illness onset [[17](#_bookmark78)]. Additionally, most are detectable by the time they are hospitalized (87% [[18](#_bookmark79)]).

Note that viral load measurements are measured in ml of serum. This is appropriate for our model, since RDTs that could potentially be used for airport screening can collect a blood sample through a finger prick [[19](#_bookmark80)].

|  | $P(\Delta N\geq x)$ | | | $P(\Delta t\geq x)$ | | |
| --- | --- | --- | --- | --- | --- | --- |
| $x$ | 7 days | 14 days | 21 days | 1 person | 10 people | 20 people |
| SARS-CoV-1 | 0.014 | 0.001 | 0 | 0.055 | 0.001 | 0 |
| SARS-CoV-2 | 0.234 | 0.037 | 0.004 | 0.567 | 0.026 | 0 |
| Influenza A | 0.576 | 0.289 | 0.149 | 0.998 | 0.728 | 0.248 |
| Ebola | 0.093 | 0.078 | 0.061 | 0.350 | 0.006 | 0 |

Table S1: **Examples of the complementary CDF** $\boldsymbol{P}(X \geq x)$ **for screening effectiveness** $\Delta N$ **and** $\Delta t$**.** For $\Delta t$, we used the same scenarios as the main text: $X=100, \lambda=1$ for SARS-CoV-1, SARS-CoV-2 and influenza A, and $X=1, \lambda=1/14$ for Ebola.


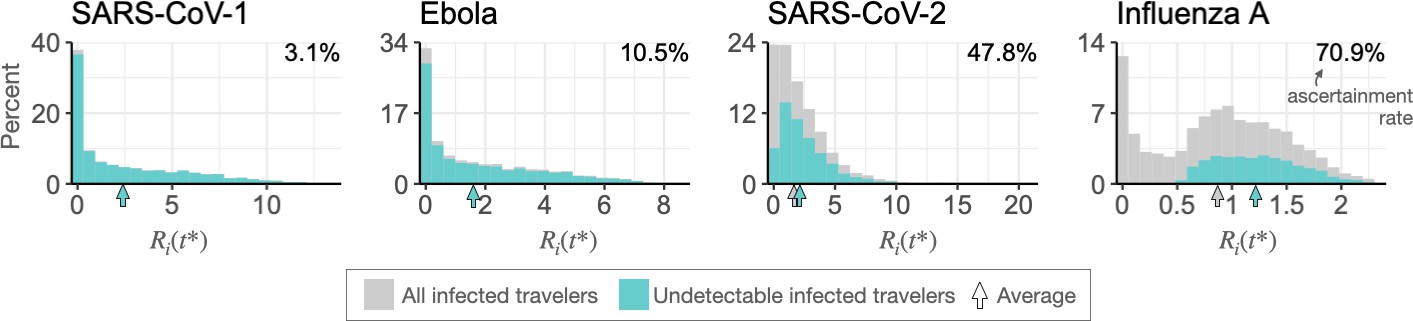


Figure S1: **Traveller screening programs decrease the number of infected travellers reaching the destination, but the average imported case has more transmission potential than without screening.** Histograms of simulated infected travellers’ transmission potential at the destination, $R_{i}(t^{*})$, with all 5000 travellers shown in grey and undetectable travellers in teal. Vertical arrows indicate the mean of each distribution. The means overlap for SARS-CoV-1 and Ebola. Ascertainment rates are reported in each upper right corner.

| **Pathogen** | **Parameter** | **Value/Distribution** | **Units** | **Source** |
| --- | --- | --- | --- | --- |
| SARS-CoV-1 | time first detectable | Unif(0,9) | days from SO | [[6](#_bookmark67), [7](#_bookmark68), [8](#_bookmark69), [9](#_bookmark70), [10](#_bookmark71), [11](#_bookmark72)] |
|  | time of peak VL | Unif(7, 14) | days from SO | [[20](#_bookmark81), [10](#_bookmark71), [7](#_bookmark68), [21](#_bookmark82)] |
|  | time last detectable | Unif(18, 28) | days from SO | [[10](#_bookmark71), [7](#_bookmark68), [8](#_bookmark69)] |
|  | PCR LOD | 2.6 | log10 copies/ml | [[22](#_bookmark83)] |
|  | infectious threshold | 6.5 | log10 copies/ml | See Methods |
|  | peak VL | Unif(5.8, 8.5) | log10 copies/ml | [[10](#_bookmark71), [23](#_bookmark84), [24](#_bookmark85), [25](#_bookmark86)] |
|  | time to symptoms | Unif(2,10) | days from infection | [[26](#_bookmark87), [27](#_bookmark88)] |
|  | time to hospitalization | Trunc. normal (*µ* = 2*.*9, *σ* = 2*.*6*, a* = *−*1*, b* = 9) | days from SO | [[26](#_bookmark87)] |
|  | $R_{0}$ | 2.55 | / | [[3](#_bookmark64)] |
|  | dispersion parameter, $k$ | 0.21 | / | [[3](#_bookmark64)] |
|  | generation interval | 8.4 | days | [[28](#_bookmark89)] |
|  | | | | |
| SARS-CoV-2  (ancestral) | time first detectable | Unif(2.6, 3.8) | days since infection | [[29](#_bookmark90)] |
|  | time of peak VL | Γ(shape=2.3, rate=0.7) | days from first detect. | [[12](#_bookmark73)] |
|  | time last detectable | Γ(shape=2.4, rate=0.3) | days from peak | [[12](#_bookmark73)] |
|  | PCR LOD | 40 | CT | [[12](#_bookmark73)] |
|  | infectious threshold | 5 | log10 copies/ml | [[30](#_bookmark91), [12](#_bookmark73)] |
|  | peak VL | Normal(*µ* = 22*.*3*, σ* = 4*.*2) | CT | [[12](#_bookmark73)] |
|  | $R_{0}$ | 2.8 | / | [[31](#_bookmark92)] |
|  | dispersion parameter, $k$ | 0.55 | / | [[32](#_bookmark93)] |
|  | generation interval | 5.9 | days | [[33](#_bookmark94)] |
|  | | | | |
| Influenza A | time first detectable | Unif(0.5, 1.5) | days from infection | [[34](#_bookmark95), [35](#_bookmark96)] |
|  | time of peak VL | Unif(1, 3) | days from first detect. | [[34](#_bookmark95), [35](#_bookmark96), [36](#_bookmark97)] |
|  | time last detectable | Unif(2, 3) | days from peak | [[34](#_bookmark95), [35](#_bookmark96), [36](#_bookmark97)] |
|  | PCR LOD | 2.95 | log10 copies/ml | [[36](#_bookmark97)] |
|  | infectious threshold | 4 | log10 copies/ml | [[37](#_bookmark98)] |
|  | peak VL | Unif(6, 8.5) | log10 copies/ml | [[36](#_bookmark97)] |
|  | $R_{0}$ | 1.26 | / | [[38](#_bookmark99)] |
|  | dispersion parameter, $k$ | 2.36 | / | [[38](#_bookmark99)] |
|  | generation interval | 2.6 | days | [[39](#_bookmark100)] |
|  | | | | |
| Ebola | time first detectable | Unif(0, 3) | days from SO | [[40](#_bookmark101), [41](#_bookmark102), [42](#_bookmark103)] |
|  | time of peak VL | Unif(3, 6) | days from SO | [[43](#_bookmark104), [44](#_bookmark105), [45](#_bookmark106), [46](#_bookmark107)] |
|  | time last detectable | Trunc. normal(*µ* = 12.7, *σ* = 3*.*8*, a* = 9*, b* = 16*.*5) | days from SO | [[43](#_bookmark104)] |
|  | PCR LOD | 2.7 | log10 copies/ml | [[45](#_bookmark106)] |
|  | infectious threshold | 7 | log10 copies/ml | See Methods |
|  | peak VL | Unif(6.5, 9.2) | log10 copies/ml | [[43](#_bookmark104), [44](#_bookmark105), [46](#_bookmark107)] |
|  | time to dry symptoms | Unif(5, 13) | days from infection | [[47](#_bookmark108), [48](#_bookmark109)] |
|  | time to wet symptoms | Unif(3, 5) | days from SO | [[41](#_bookmark102)] |
|  | time to hospitalization | Trunc. normal(*µ* = 4.5,  *σ* = 2*.*5*, a* = 2*, b* = 7) | days from SO | [[43](#_bookmark104)] |
|  | $R_{0}$ | 1.8 | / | [[49](#_bookmark110), [50](#_bookmark111), [51](#_bookmark112)] |
|  | dispersion parameter, $k$ | 0.18 | / | [[51](#_bookmark112)] |
|  | generation interval | 13 | days | [[48](#_bookmark109)] |

Table S2: **Model parameters.** Values or distributions used for each pathogen-specific parameter. SO stands for symptom onset.

| **Influenza A** | | | | | | | |
| --- | --- | --- | --- | --- | --- | --- | --- |
|  | Arrival rate ($\lambda$) | Outbreak threshold ($X$) | With screening  ($N_{0}$or $t_{0})$ | Without screening  $(N'$ or $t^{'}$) | Screening effectiveness  $(\Delta N$ or $\Delta t)$ | | % of simulations with  $\Delta t>1$ week |
|  |  |  | Mean (sd) | Mean (sd) | Mean | IQR |  |
| Number required to attempt travel to: |  |  |  |  |  |  |  |
| Likely trigger an outbreak (theory) | / | / | 9.9 (2) | 24.7 (8.2) | 14.8 | [9,19] | / |
| Generate a secondary case at the destination (simulation) | / | / | 1.9 (1.3) | 4.9 (4.4) | 3 | [0, 5] | / |
| Time to $X$ infections generated at the destination (simulation) | 1 per day | 1 | 3.2 (2.1) | 7 (5) | 3.9 | [0, 5.9] | 19.8% |
|  |  | 10 | 8.1 (3.2) | 15.1 (7.8) | 6.9 | [2.2, 9.6] | 36.1% |
|  |  | 100 | 20.2 (5.3) | 31.6 (11.7) | 11.4 | [4.2, 15.6] | 57.6% |
|  | 1 per week | 1 | 14.7 (13.4) | 36.7 (34.7) | 22 | [0, 32.4] | 51.4% |
|  |  | 10 | 30.8 (22.3) | 68.2 (56.1) | 37.4 | [0.1, 56.0] | 58.8% |
|  |  | 100 | 28.3 (27.2) | 71.3 (69.1) | 43 | [2.6, 71.6] | 56.4% |
|  | 2 per month | 1 | 28.3 (27.2) | 71.3 (69.1) | 43 | [0, 61.7] | 56.4% |
|  |  | 10 | 54.9 (45.1) | 128.4 (113) | 73.5 | [0, 109.2] | 61.2% |
|  |  | 100 | 89.3 (64) | 185 (158.3) | 95.8 | [0, 136.3] | 61.1% |

Figure S2: **Screening effectiveness for influenza A.** Screening effectiveness quantified by comparing the difference between the number of infected travellers required to attempt travel to likely generate an outbreak and the time to $X$ infections generated at the destination, with and without screening. The grey row is the plausible example reported in the Main Text.

| **SARS-CoV-2** | | | | | | | |
| --- | --- | --- | --- | --- | --- | --- | --- |
|  | Arrival rate ($\lambda$) | Outbreak threshold ($X$) | With screening  ($N_{0}$or $t_{0})$ | Without screening  $(N'$ or $t^{'}$) | Screening effectiveness  $(\Delta N$ or $\Delta t)$ | | % of simulations with  $\Delta t>1$ week |
|  |  |  | Mean (sd) | Mean (sd) | Mean | IQR |  |
| Number required to attempt travel to: |  |  |  |  |  |  |  |
| Likely trigger an outbreak (theory) | / | / | 3.1 (1.5) | 5.2 (3.1) | 2.1 | [0, 3] | / |
| Generate a secondary case at the destination (simulation) | / | / | 1.5 (0.9) | 2.5 (2) | 1 | [0, 1] | / |
| Time to $X$ infections generated at the destination (simulation) | 1 per day | 1 | 4.6 (2.5) | 7 (2.7) | 2.4 | [0, 5.0] | 11.6% |
|  |  | 10 | 8 (3) | 12 (4) | 4 | [0.6, 5.9] | 18.1% |
|  |  | 100 | 17.4 (3.5) | 22.2 (4.7) | 4.8 | [1.5, 6.7] | 23.4% |
|  | 1 per week | 1 | 13.6 (10.7) | 22.3 (18) | 8.6 | [0, 11.8] | 33.1% |
|  |  | 10 | 23.4 (13.9) | 34.3 (22.6) | 10.9 | [0, 14.5] | 35.6% |
|  |  | 100 | 35.7 (14.9) | 47.3 (23.7) | 11.6 | [0, 15.4] | 36.6% |
|  | 2 per month | 1 | 24.1 (21) | 39.8 (35.4) | 15.7 | [0, 20.5] | 36.3% |
|  |  | 10 | 37.3 (26.6) | 57.2 (44.8) | 19.9 | [0, 25.3] | 37.8% |
|  |  | 100 | 51.2 (28.6) | 71.6 (46.3) | 20.4 | [0, 26.1] | 38.7% |

Figure S3: **Screening effectiveness for SARS-CoV-2.** Screening effectiveness quantified by comparing the difference between the number of infected travellers required to attempt travel to likely generate an outbreak and the time to $X$ infections generated at the destination, with and without screening. The grey row is the plausible example reported in the Main Text.

| **Ebola with infectious threshold = 7 (value used in the main text)** | | | | | | | |
| --- | --- | --- | --- | --- | --- | --- | --- |
|  | Arrival rate ($\lambda$) | Outbreak threshold ($X$) | With screening  ($N_{0}$or $t_{0})$ | Without screening  $(N'$ or $t^{'}$) | Screening effectiveness  $(\Delta N$ or $\Delta t)$ | | % of simulations with  $\Delta t>1$ week |
|  |  |  | Mean (sd) | Mean (sd) | Mean | IQR |  |
| Number required to attempt travel to: |  |  |  |  |  |  |  |
| Likely trigger an outbreak (theory) | / | / | 9.5 (3.4) | 10.6 (3.9) | 1.1 | [0, 1] | / |
| Generate a secondary case at the destination (simulation) | / | / | 1.9 (1.3) | 2.1 (1.5) | 0.2 | [0, 0] | / |
| Time to $X$ infections generated at the destination (simulation) | 1 per day | 1 | 10.1 (4.1) | 11 (3.8) | 0.9 | [0, 0] | 6.8% |
|  |  | 10 | 14.6 (4.5) | 16.1 (4.7) | 1.5 | [0, 1.8] | 6.7% |
|  |  | 100 | 36.5 (6.7) | 38.8 (7.2) | 2.3 | [0, 3.3] | 10.2% |
|  | 1 per week | 1 | 21.6 (13.8) | 23.8 (15.1) | 2.1 | [0, 0] | 9.1% |
|  |  | 10 | 41.7 (21.6) | 45.4 (23.8) | 3.7 | [0, 0] | 14.1% |
|  |  | 100 | 84.8 (34.8) | 90 (38) | 5.2 | [0, 0] | 15.7% |
|  | 2 per month | 1 | 34.7 (26.6) | 38.3 (29.6) | 3.5 | [0, 0] | 9.2% |
|  |  | 10 | 68.1 (43.3) | 74.3 (48.2) | 6.2 | [0, 0] | 13.7% |
|  |  | 100 | 122.5 (66.3) | 130.8 (73.3) | 8.3 | [0, 0] | 14.4% |

Figure S4: **Screening effectiveness for Ebola.** Screening effectiveness quantified by comparing the difference between the number of infected travellers required to attempt travel to likely generate an outbreak and the time to $X$ infections generated at the destination, with and without screening. The grey row is the plausible example reported in the Main Text.

| **SARS-CoV-1 with infectious threshold = 6.5 (value used in the main text)** | | | | | | | |
| --- | --- | --- | --- | --- | --- | --- | --- |
|  | Arrival rate ($\lambda$) | Outbreak threshold ($X$) | With screening  ($N_{0}$or $t_{0})$ | Without screening  $(N'$ or $t^{'}$) | Screening effectiveness  $(\Delta N$ or $\Delta t)$ | | % of simulations with  $\Delta t>1$ week |
|  |  |  | Mean (sd) | Mean (sd) | Mean | IQR |  |
| Number required to attempt travel to: |  |  |  |  |  |  |  |
| Likely trigger an outbreak (theory) | / | / | 4.7 (2.6) | 4.9 (2.7) | 0.2 | [0, 0] | / |
| Generate a secondary case at the destination (simulation) | / | / | 1.9 (1.3) | 1.9 (1.3) | 0.1 | [0, 0] | / |
| Time to $X$ infections generated at the destination (simulation) | 1 per day | 1 | 7.5 (3) | 7.7 (3) | 0.2 | [0, 0] | 1.4% |
|  |  | 10 | 10.4 (3.8) | 10.8 (3.8) | 0.3 | [0, 0] | 1.4% |
|  |  | 100 | 23.1 (4.8) | 23.6 (4.9) | 0.5 | [0, 0] | 1.4% |
|  | 1 per week | 1 | 18.7 (13.2) | 19.2 (13.6) | 0.5 | [0, 0] | 2.5% |
|  |  | 10 | 30.2 (18) | 31 (18.5) | 0.8 | [0, 0] | 3.4% |
|  |  | 100 | 50 (21.8) | 50.9 (22.3) | 0.9 | [0, 0] | 3.6% |
|  | 2 per month | 1 | 31.7 (25.9) | 32.5 (26.6) | 0.8 | [0, 0] | 2.6% |
|  |  | 10 | 50 (36) | 51.4 (37.2) | 1.4 | [0, 0] | 3.5% |
|  |  | 100 | 72.1 (41.2) | 73.6 (42.4) | 1.5 | [0, 0] | 3.6% |

Figure S5: **Screening effectiveness for SARS-CoV-1.** Screening effectiveness quantified by comparing the difference between the number of infected travellers required to attempt travel to likely generate an outbreak and the time to $X$ infections generated at the destination, with and without screening. The grey row is the plausible example reported in the Main Text.


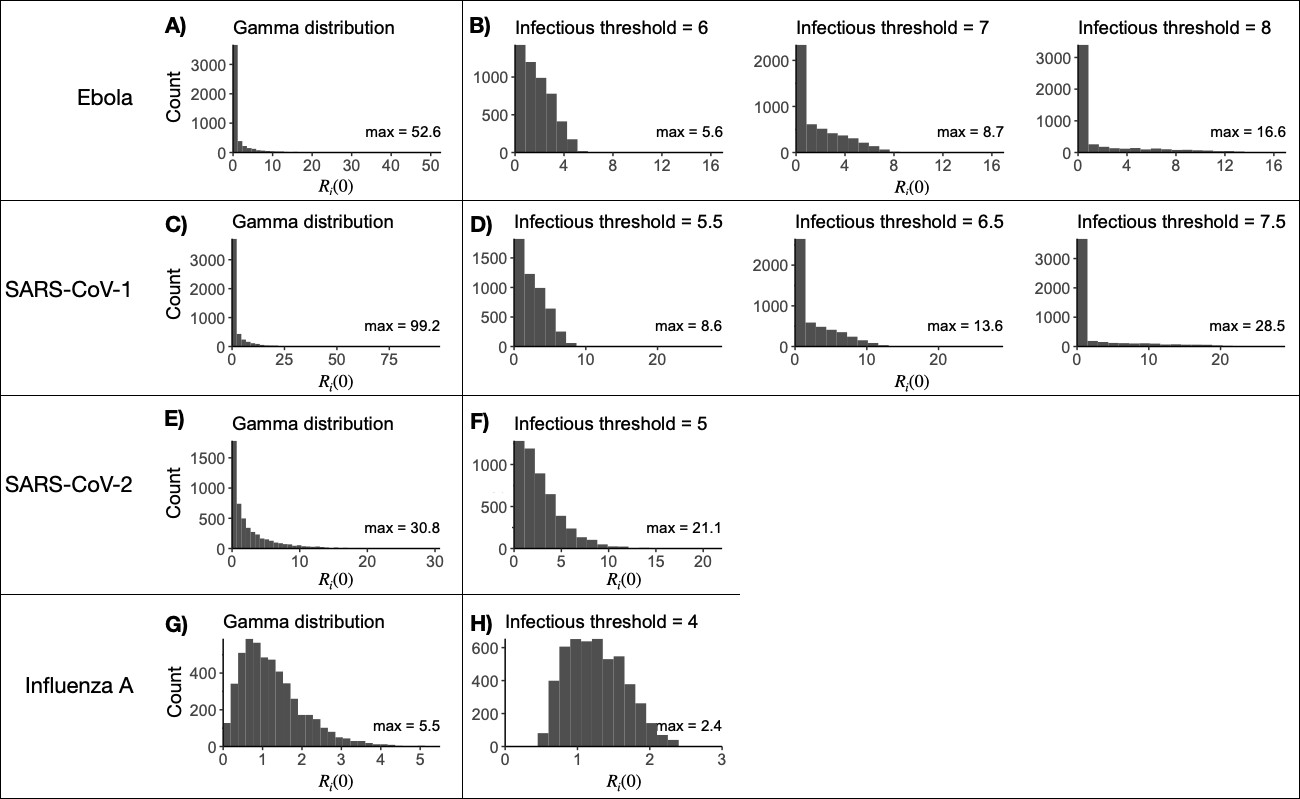


Figure S6: **Different approaches to calculate the individual reproductive number** $\boldsymbol{R}_{\boldsymbol{i}}\boldsymbol{(0)}$ **result in important differences in the population-level distributions of** $\boldsymbol{R}_{\boldsymbol{i}}\boldsymbol{(0)}$**. (A, C, E, G)** Distribution of the individual reproductive number $R_{i}(0)$ sampled from a gamma distribution with mean *R*_0_ and dispersion parameter *k*. **(B, D, F, H)** Distribution of $R_{i}(0)$ from the within-host viral kinetics model with various infectious thresholds.

| **Ebola with infectious threshold = 6** | | | | | | | |
| --- | --- | --- | --- | --- | --- | --- | --- |
|  | Arrival rate ($\lambda$) | Outbreak threshold ($X$) | With screening  ($N_{0}$or $t_{0})$ | Without screening  $(N'$ or $t^{'}$) | Screening effectiveness  $(\Delta N$ or $\Delta t)$ | | % of simulations with  $\Delta t>1$ week |
|  |  |  | Mean (sd) | Mean (sd) | Mean | IQR |  |
| Number required to attempt travel to: |  |  |  |  |  |  |  |
| Likely trigger an outbreak (theory) | / | / | 9.1 (2) | 10 (2.5) | 0.9 | [0, 1] | / |
| Generate a secondary case at the destination (simulation) | / | / | 1.4 (0.8) | 1.6 (1) | 0.2 | [0, 0] | / |
| Time to $X$ infections generated at the destination (simulation) | 1 per day | 1 | 9.7 (3.9) | 10.5 (3.5) | 0.8 | [0, 0] | 6.4% |
|  |  | 10 | 14.3 (3.7) | 15.8 (3.8) | 1.5 | [0, 2.1] | 4.9% |
|  |  | 100 | 36.1 (5.9) | 38.5 (6.4) | 2.3 | [0, 3.4] | 9.5% |
|  | 1 per week | 1 | 18.2 (10.6) | 19.9 (11.4) | 1.7 | [0, 0] | 8.4% |
|  |  | 10 | 39.5 (17.8) | 42.8 (19.6) | 3.3 | [0, 0] | 14.5% |
|  |  | 100 | 82.4 (31.2) | 87.6 (34.6) | 5.1 | [0, 0.4] | 16.7% |
|  | 2 per month | 1 | 27.9 (20.1) | 30.7 (22.1) | 2.8 | [0, 0] | 9.2% |
|  |  | 10 | 61.9 (35.4) | 67.2 (39) | 5.3 | [0, 0] | 15.1% |
|  |  | 100 | 117.7 (59.8) | 125.6 (66) | 7.9 | [0, 0] | 14.9% |

Figure S7: **Screening effectiveness for Ebola with a lower infectious threshold.** Screening effectiveness quantified by comparing the difference between the number of infected travellers required to attempt travel to likely generate an outbreak and the time to $X$ infections generated at the destination, with and without screening. The infectious threshold is set to 6 log10 copies RNA/ml. An infectious threshold of 7 log10 copies RNA/ml was used in the Main Text.

| **Ebola with infectious threshold = 8** | | | | | | | |
| --- | --- | --- | --- | --- | --- | --- | --- |
|  | Arrival rate ($\lambda$) | Outbreak threshold ($X$) | With screening  ($N_{0}$or $t_{0})$ | Without screening  $(N'$ or $t^{'}$) | Screening effectiveness  $(\Delta N$ or $\Delta t)$ | | % of simulations with  $\Delta t>1$ week |
|  |  |  | Mean (sd) | Mean (sd) | Mean | IQR |  |
| Number required to attempt travel to: |  |  |  |  |  |  |  |
| Likely trigger an outbreak (theory) | / | / | 10.6 (5.8) | 11.8 (6.5) | 1.1 | [0, 0] | / |
| Generate a secondary case at the destination (simulation) | / | / | 3.1 (2.6) | 3.4 (2.9) | 0.3 | [0, 0] | / |
| Time to $X$ infections generated at the destination (simulation) | 1 per day | 1 | 11.5 (4.8) | 12.4 (4.7) | 0.9 | [0, 0] | 6.8% |
|  |  | 10 | 15.5 (6.1) | 16.9 (6.4) | 1.5 | [0, 0] | 8.2% |
|  |  | 100 | 37.2 (8.3) | 39.4 (8.9) | 2.2 | [0, 2.9] | 11.5% |
|  | 1 per week | 1 | 30.2 (22) | 33.1 (24) | 2.9 | [0, 0] | 9.1% |
|  |  | 10 | 48.3 (31.3) | 52.6 (34.8) | 4.2 | [0, 0] | 11.6% |
|  |  | 100 | 90.7 (42.6) | 96.4 (46.9) | 5.7 | [0, 0] | 14.3% |
|  | 2 per month | 1 | 52.3 (44.1) | 57.5 (48.8) | 5.2 | [0, 0] | 9.2% |
|  |  | 10 | 82.2 (62.1) | 89.6 (68.7) | 7.3 | [0, 0] | 11.4% |
|  |  | 100 | 135.9 (82.1) | 145.6 (90.7) | 9.7 | [0, 0] | 13.1% |

Figure S8: **Screening effectiveness for Ebola with a higher infectious threshold.** Screening effectiveness quantified by comparing the difference between the number of infected travellers required to attempt travel to likely generate an outbreak and the time to $X$ infections generated at the destination, with and without screening. The infectious threshold is 8 log10 copies RNA/ml. An infectious threshold of 7 log10 copies RNA/ml was used in the Main Text.

| **SARS-CoV-1 with infectious threshold = 5.5** | | | | | | | |
| --- | --- | --- | --- | --- | --- | --- | --- |
|  | Arrival rate ($\lambda$) | Outbreak threshold ($X$) | With screening  ($N_{0}$or $t_{0})$ | Without screening  $(N'$ or $t^{'}$) | Screening effectiveness  $(\Delta N$ or $\Delta t)$ | | % of simulations with  $\Delta t>1$ week |
|  |  |  | Mean (sd) | Mean (sd) | Mean | IQR |  |
| Number required to attempt travel to: |  |  |  |  |  |  |  |
| Likely trigger an outbreak (theory) | / | / | 4.3 (1.5) | 4.4 (1.6) | 0.1 | [0, 0] | / |
| Generate a secondary case at the destination (simulation) | / | / | 1.3 (0.6) | 1.4 (0.7) | 0 | [0, 0] | / |
| Time to $X$ infections generated at the destination (simulation) | 1 per day | 1 | 6.9 (2.7) | 7.1 (2.6) | 0.2 | [0, 0] | 1.1% |
|  |  | 10 | 10.2 (3) | 10.4 (3) | 0.3 | [0, 0] | 0.5% |
|  |  | 100 | 22.9 (4.1) | 23.2 (4.2) | 0.4 | [0, 0] | 0.7% |
|  | 1 per week | 1 | 14.9 (9.6) | 15.3 (9.8) | 0.4 | [0, 0] | 1.9% |
|  |  | 10 | 27.8 (14.2) | 28.4 (14.5) | 0.6 | [0, 0] | 2.7% |
|  |  | 100 | 27.8 (14.2) | 28.4 (14.5) | 0.7 | [0, 0] | 3% |
|  | 2 per month | 1 | 24.2 (18.6) | 24.8 (19.1) | 0.6 | [0, 0] | 2.2% |
|  |  | 10 | 43.5 (28.1) | 44.5 (28.9) | 1 | [0, 0] | 3.3% |
|  |  | 100 | 66.9 (35) | 67.9 (35.7) | 1 | [0, 0] | 3% |

Figure S9: **Screening effectiveness for SARS-CoV-1 with a lower infectious threshold.** Screening effectiveness quantified by comparing the difference between the number of infected travellers required to attempt travel to likely generate an outbreak and the time to $X$ infections generated at the destination, with and without screening. The infectious threshold is 5.5 log10 copies RNA/ml. An infectious threshold of 6.5 log10 copies RNA/ml was used in the Main Text.

| **SARS-CoV-1 with infectious threshold = 7.5** | | | | | | | |
| --- | --- | --- | --- | --- | --- | --- | --- |
|  | Arrival rate ($\lambda$) | Outbreak threshold ($X$) | With screening  ($N_{0}$or $t_{0})$ | Without screening  $(N'$ or $t^{'}$) | Screening effectiveness  $(\Delta N$ or $\Delta t)$ | | % of simulations with  $\Delta t>1$ week |
|  |  |  | Mean (sd) | Mean (sd) | Mean | IQR |  |
| Number required to attempt travel to: |  |  |  |  |  |  |  |
| Likely trigger an outbreak (theory) | / | / | 6.2 (4.9) | 6.4 (5.1) | 0.2 | [0,0] | / |
| Generate a secondary case at the destination (simulation) | / | / | 3.4 (2.9) | 3.5 (3) | 0.1 | [0,0] | / |
| Time to $X$ infections generated at the destination (simulation) | 1 per day | 1 | 9.1 (4.2) | 9.3 (4.2) | 0.2 | [0,0] | 1.55% |
|  |  | 10 | 11.5 (5.5) | 11.8 (5.6) | 0.3 | [0,0] | 1.54% |
|  |  | 100 | 23.9 (6.5) | 24.3 (6.7) | 0.3 | [0,0] | 1.52% |
|  | 1 per week | 1 | 30 (24.4) | 30.8 (25) | 0.7 | [0,0] | 2.26% |
|  |  | 10 | 39.7 (30.2) | 40.5 (30.9) | 0.8 | [0,0] | 2.39% |
|  |  | 100 | 58.5 (32.1) | 59.5 (33) | 1 | [0,0] | 2.94% |
|  | 2 per month | 1 | 54 (48.4) | 55.4 (49.4) | 1.3 | [0,0] | 2.36% |
|  |  | 10 | 70.7 (58.9) | 72.5 (60.5) | 1.8 | [0,0] | 2.83% |
|  |  | 100 | 91.7 (63.8) | 93.2 (65) | 1.5 | [0,0] | 2.5% |

Figure S10: **Screening effectiveness for SARS-CoV-1 with a higher infectious threshold.** Screening effectiveness quantified by comparing the difference between the number of infected travellers required to attempt travel to likely generate an outbreak and the time to $X$ infections generated at the destination, with and without screening. The infectious threshold is 7.5 log10 copies RNA/ml. An infectious threshold of 6.5 log10 copies RNA/ml was used in the Main Text.

| **Ebola with infectious threshold = 7 (value used in the main text),** $\boldsymbol{D =}\boldsymbol{t}_{\boldsymbol{clearance}}$ | | | | | | | |
| --- | --- | --- | --- | --- | --- | --- | --- |
|  | Arrival rate ($\lambda$) | Outbreak threshold ($X$) | With screening  ($N_{0}$or $t_{0})$ | Without screening  $(N'$ or $t^{'}$) | Screening effectiveness  $(\Delta N$ or $\Delta t)$ | | % of simulations with  $\Delta t>1$ week |
|  |  |  | Mean (sd) | Mean (sd) | Mean | IQR |  |
| Number required to attempt travel to: |  |  |  |  |  |  |  |
| Likely trigger an outbreak (theory) | / | / | 11.6 (4.3) | 13.9 (5.5) | 2.3 | [0, 4] | / |
| Generate a secondary case at the destination (simulation) | / | / | 2.2 (1.7) | 2.7 (2.1) | 0.5 | [0, 0] | / |
| Time to $X$ infections generated at the destination (simulation) | 1 per day | 1 | 9.5 (4.5) | 11.1 (4.2) | 1.6 | [0, 0] | 12.1% |
|  |  | 10 | 15 (5.2) | 17.6 (5.7) | 2.6 | [0, 4.2] | 13.9% |
|  |  | 100 | 38.5 (7.7) | 42.6 (8.9) | 4.1 | [0, 6.4] | 22.2% |
|  | 1 per week | 1 | 23.2 (16.3) | 27.5 (19.1) | 4.3 | [0, 0] | 15.7% |
|  |  | 10 | 46.5 (26) | 53.8 (31.3) | 7.3 | [0, 4.8] | 22.9% |
|  |  | 100 | 92.5 (41.5) | 103.1 (49.9) | 10.5 | [0, 7.6] | 25.4% |
|  | 2 per month | 1 | 38.7 (31.7) | 46.6 (38.7) | 7.9 | [0, 0] | 16.5% |
|  |  | 10 | 76.8 (52.1) | 89.2 (62.4) | 12.4 | [0, 1.5] | 22.4% |
|  |  | 100 | 137.1 (79.3) | 154.2 (93.8) | 17.1 | [0, 2.9] | 23.2% |

Figure S11: **Screening effectiveness** **for Ebola assuming people travel until viral clearance.** Screening effectiveness quantified by comparing the difference between the number of infected travellers required to attempt travel to likely generate an outbreak and the time to $X$ infections generated at the destination, with and without screening. Using the notation from the Methods section and Figure 1, $D=t_{4}= t_{clearance}$ (i.e., time last detectable in Table S2). In the Main Text, we assumed people infected with Ebola virus travelled up until the time they were hospitalized (i.e., $D=t_{hosp})$.

| **SARS-CoV-1 with infectious threshold = 6.5 (value used in the main text),** $\boldsymbol{D =}\boldsymbol{t}_{\boldsymbol{clearance}}$ | | | | | | | |
| --- | --- | --- | --- | --- | --- | --- | --- |
|  | Arrival rate ($\lambda$) | Outbreak threshold ($X$) | With screening  ($N_{0}$or $t_{0})$ | Without screening  $(N'$ or $t^{'}$) | Screening effectiveness  $(\Delta N$ or $\Delta t)$ | | % of simulations with  $\Delta t>1$ week |
|  |  |  | Mean (sd) | Mean (sd) | Mean | IQR |  |
| Number required to attempt travel to: |  |  |  |  |  |  |  |
| Likely trigger an outbreak (theory) | / | / | 5.7 (3.2) | 7.6 (4.6) | 1.9 | [0,3] | / |
| Generate a secondary case at the destination (simulation) | / | / | 2.1 (1.5) | 2.9 (2.3) | 0.8 | [0,1] | / |
| Time to $X$ infections generated at the destination (simulation) | 1 per day | 1 | 5.2 (3.6) | 7 (4) | 1.8 | [0, 0.5] | 12.5 |
|  |  | 10 | 8.6 (4.1) | 11.3 (5) | 2.7 | [0, 4.5] | 14.5 |
|  |  | 100 | 22 (5.3) | 25.8 (6.5) | 3.8 | [0, 6.0] | 20.5 |
|  | 1 per week | 1 | 18.1 (15.3) | 24.3 (20.6) | 6.2 | [0, 1.2] | 21.9 |
|  |  | 10 | 31.1 (21.2) | 39.6 (28) | 8.5 | [0, 8.4] | 27.5 |
|  |  | 100 | 51.7 (25) | 61.4 (32.6) | 9.7 | [0, 8.4] | 28.2 |
|  | 2 per month | 1 | 32.8 (29.9) | 44.3 (40.5) | 11.5 | [0, 4.1] | 23.6 |
|  |  | 10 | 52.9 (41) | 68.9 (55.4) | 16 | [0, 8.4] | 28.7 |
|  |  | 100 | 76.7 (48.1) | 94.3 (63.5) | 17.6 | [0, 8.4] | 28.4 |

Figure S12: **Screening effectiveness for SARS-CoV-1 assuming people travel until viral clearance.** Screening effectiveness quantified by comparing the difference between the number of infected travellers required to attempt travel to likely generate an outbreak and the time to $X$ infections generated at the destination, with and without screening. Using the notation from the Methods section and Figure 1, $D=t_{4}= t_{clearance}$ (i.e., time last detectable in Table S2). In the Main Text, we assumed people infected with SARS-CoV-1 travelled up until the time they were hospitalized (i.e., $D=t_{hosp})$.


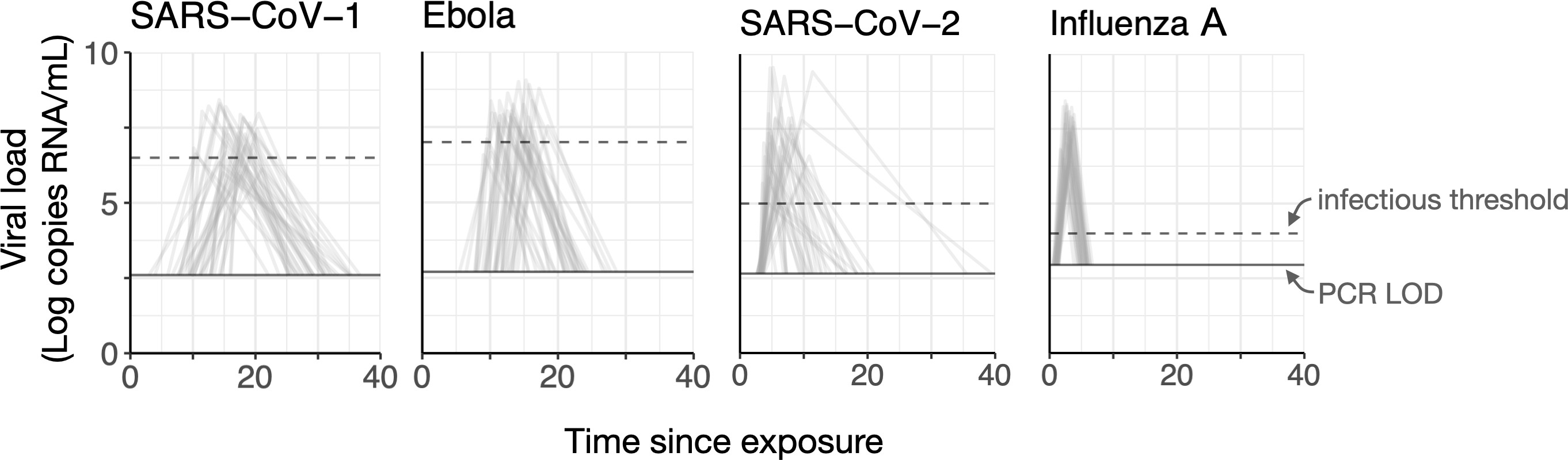


Figure S13: **Simulated viral load trajectories for SARS-CoV-1, Ebola, SARS-CoV-2 and influenza A.** 100 stochastically drawn viral load trajectories for SARS-CoV-1, Ebola, SARS-CoV- 2, and influenza A, using the control points and parameter values in Table [S2](#_bookmark51).

**References**

1. Clifford, S et al. (2020). Effectiveness of interventions targeting air travellers for delaying local outbreaks of SARS-CoV-2. Journal of Travel Medicine. 27: taaa068.
2. Gostic, KM, Kucharski, AJ, and Lloyd-Smith, JO (2015). Effectiveness of traveller screening for emerging pathogens is shaped by epidemiology and natural history of infection. eLife. 4: e05564.
3. Lloyd-Smith, JO et al. (2005). Superspreading and the effect of individual variation on disease emergence. Nature. 438: 355–359.
4. Hui, DS and Zumla, A (2019). Severe acute respiratory syndrome: historical, epidemiologic, and clinical features. Infectious Disease Clinics. 33: 869–889.
5. Wilder-Smith, A et al. (2005). Asymptomatic SARS coronavirus infection among healthcare workers, Singapore. Emerging Infectious Diseases. 11: 1142.
6. Poon, LL et al. (2004). Detection of SARS coronavirus in patients with severe acute respiratory syndrome by conventional and real-time quantitative reverse transcription-PCR assays. Clinical Chemistry. 50: 67–72.
7. Cheng, PK et al. (2004). Viral shedding patterns of coronavirus in patients with probable severe acute respiratory syndrome. The Lancet. 363: 1699–1700.
8. Chan, KH et al. (2004). Detection of SARS coronavirus in patients with suspected SARS. Emerging Infectious Diseases. 10: 294.
9. Chu, C et al. (2005). Duration of RT-PCR positivity in severe acute respiratory syndrome. European Respiratory Journal. 25: 12–14.
10. Peiris, JSM et al. (2003). Clinical progression and viral load in a community outbreak of coronavirus-associated SARS pneumonia: a prospective study. The Lancet. 361: 1767–1772.
11. Chan, PK et al. (2004). Laboratory diagnosis of SARS. Emerging Infectious Diseases. 10: 825.
12. Kissler, SM et al. (2021). Viral dynamics of acute SARS-CoV-2 infection and applications to diagnostic and public health strategies. PLoS Biology. 19: e3001333.
13. Hartley, M.-A et al. (2017). Predicting Ebola severity: a clinical prioritization score for Ebola virus disease. PLoS Neglected Tropical Diseases. 11: e0005265.
14. Stadler, T et al. (2014). Insights into the early epidemic spread of Ebola in Sierra Leone provided by viral sequence data. PLoS Currents. 6.
15. Vel´asquez, GE et al. (2015). Time from infection to disease and infectiousness for Ebola virus disease, a systematic review. Clinical Infectious Diseases. 61: 1135–1140.
16. Kuhn, JH and Bavari, S (2017). Asymptomatic Ebola virus infections—myth or reality? The Lancet Infectious Diseases. 17: 570–571.
17. Kortepeter, MG, Bausch, DG, and Bray, M (2011). Basic clinical and laboratory features of filoviral hemorrhagic fever. The Journal of Infectious Diseases. 204: S810–S816.
18. Hunt, L et al. (2015). Clinical presentation, biochemical, and haematological parameters and their association with outcome in patients with Ebola virus disease: an observational cohort study. The Lancet Infectious Diseases. 15: 1292–1299.
19. Nouvellet, P et al. (2015). The role of rapid diagnostics in managing Ebola epidemics. Nature. 528: S109–S116.
20. Anderson, RM et al. (2004). Epidemiology, transmission dynamics and control of SARS: the 2002–2003 epidemic. Philosophical Transactions of the Royal Society of London. Series B: Biological Sciences. 359: 1091–1105.
21. Cevik, M et al. (2021). SARS-CoV-2, SARS-CoV, and MERS-CoV viral load dynamics, dura- tion of viral shedding, and infectiousness: a systematic review and meta-analysis. The Lancet Microbe. 2: e13–e22.
22. Hourfar, MK et al. (2004). Comparison of two real-time quantitative assays for detection of severe acute respiratory syndrome coronavirus. Journal of Clinical Microbiology. 42: 2094– 2100.
23. Hung, I et al. (2004). Viral loads in clinical specimens and SARS manifestations. Emerging Infectious Diseases. 10: 1550.
24. Chen, W.-J et al. (2006). Nasopharyngeal shedding of severe acute respiratory syndrome—associated coronavirus is associated with genetic polymorphisms. Clinical Infectious Diseases. 42: 1561–1569.
25. Drosten, C et al. (2003). Identification of a novel coronavirus in patients with severe acute respiratory syndrome. New England Journal of Medicine. 348: 1967–1976.
26. Leung, GM et al. (2004). The epidemiology of severe acute respiratory syndrome in the 2003 Hong Kong epidemic: an analysis of all 1755 patients. Annals of Internal Medicine. 141: 662– 673.
27. Donnelly, CA et al. (2003). Epidemiological determinants of spread of causal agent of severe acute respiratory syndrome in Hong Kong. The Lancet. 361: 1761–1766.
28. Lipsitch, M et al. (2003). Transmission dynamics and control of severe acute respiratory syndrome. Science. 300: 1966–1970.
29. Puhach, O, Meyer, B, and Eckerle, I (2023). SARS-CoV-2 viral load and shedding kinetics. Nature Reviews Microbiology. 21: 147–161.
30. Gallichotte, E et al. (2021). Early adoption of longitudinal surveillance for SARS-CoV-2 among staff in long-term care facilities: prevalence, virologic and sequence analysis. Spectrum. 9: 01003–21.
31. Liu, Y et al. (2020). The reproductive number of COVID-19 is higher compared to SARS coronavirus. Journal of Travel Medicine. 27: taaa021.
32. Du, Z et al. (2022). Systematic review and meta-analyses of superspreading of SARS-CoV-2 infections. Transboundary and Emerging Diseases. 69: e3007–e3014.
33. Hart, WS et al. (2022). Inference of the SARS-CoV-2 generation time using UK household data. eLife. 11: e70767.
34. Carrat, F et al. (2008). Time lines of infection and disease in human influenza: a review of volunteer challenge studies. American Journal of Epidemiology. 167: 775–785.
35. Sloan, SE et al. (2020). Clinical and virological responses to a broad-spectrum human mon- oclonal antibody in an influenza virus challenge study. Antiviral Research. 184: 104763.
36. Ip, DK et al. (2016). The dynamic relationship between clinical symptomatology and viral shedding in naturally acquired seasonal and pandemic influenza virus infections. Clinical Infectious Diseases. 62: 431–437.
37. Lau, LL et al. (2010). Viral shedding and clinical illness in naturally acquired influenza virus infections. The Journal of Infectious Diseases. 201: 1509–1516.
38. Goyal, A et al. (2021). Viral load and contact heterogeneity predict SARS-CoV-2 transmission and super-spreading events. eLife. 10: e63537.
39. Beest, DE te et al. (2013). Estimating the generation interval of influenza A (H1N1) in a range of social settings. Epidemiology. 244–250.
40. Broadhurst, MJ, Brooks, TJ, and Pollock, NR (2016). Diagnosis of Ebola virus disease: past, present, and future. Clinical Microbiology Reviews. 29: 773–793.
41. Chertow, DS et al. (2014). Ebola virus disease in West Africa—clinical manifestations and management. New England Journal of Medicine. 371: 2054–2057.
42. Jacob, ST et al. (2020). Ebola virus disease. Nature Reviews Disease Primers. 6: 13.
43. Lanini, S et al. (2015). Blood kinetics of Ebola virus in survivors and nonsurvivors. The Journal of Clinical Investigation. 125: 4692–4698.
44. Towner, JS et al. (2004). Rapid diagnosis of Ebola hemorrhagic fever by reverse transcription- PCR in an outbreak setting and assessment of patient viral load as a predictor of outcome. Journal of Virology. 78: 4330–4341.
45. Matson, MJ et al. (2022). Evaluation of viral load in patients with Ebola virus disease in Liberia: a retrospective observational study. The Lancet Microbe. 3: e533–e542.
46. Schibler, M et al. (2015). Clinical features and viral kinetics in a rapidly cured patient with Ebola virus disease: a case report. The Lancet Infectious Diseases. 15: 1034–1040.
47. Van Kerkhove, MD et al. (2015). A review of epidemiological parameters from Ebola outbreaks to inform early public health decision-making. Scientific Data. 2: 1–10.
48. Team, WER (2015). West African Ebola epidemic after one year—slowing but not yet under control. New England Journal of Medicine. 372: 584–587.
49. Fisman, D, Khoo, E, and Tuite, A (2014). Early epidemic dynamics of the West African 2014 Ebola outbreak: estimates derived with a simple two-parameter model. PLoS Currents. 6.
50. Gomes, MF et al. (2014). Assessing the international spreading risk associated with the 2014 West African Ebola outbreak. PLoS Currents. 6.
51. Althaus, CL (2014). Estimating the reproduction number of Ebola virus (EBOV) during the 2014 outbreak in West Africa. PLoS Currents. 6.
